# Brainstem Influence on Thalamocortical Oscillations during Anesthesia Emergence

**DOI:** 10.3389/fnsys.2017.00066

**Published:** 2017-09-14

**Authors:** Christopher M. Scheib

**Affiliations:** Anesthesia Department, W. G. (Bill) Hefner VA Medical Center Salisbury, NC, United States

**Keywords:** electroencephalogram, EEG, anesthesia, thalamus, brainstem, spindles, spectrum, emergence

## Abstract

Theories of mechanisms that impair or prevent consciousness during anesthesia that are related to thalamocortical oscillations have been proposed. Many methods of EEG analysis have been proposed as measures of anesthetic effects but only a few have potential to provide measures of those anesthetic effects that are directly related to thalamocortical oscillations. Some of these methods will be explained and demonstrated with examples chosen to provide evidence for or against two of the proposed mechanisms. The first of the two mechanisms to be addressed is the “traveling peak” (Ching et al., 2010), which relates to anesthetic agents synchronizing neural oscillations that occur in subjects who are awake and reducing their frequency from the gamma (25–40 Hz) to the beta range (13–24 Hz) as a state of sedation develops. The mechanism continues to lower the frequency of this oscillation to the alpha (8–12 Hz) range. In the alpha frequency range, responses to sounds and words stop. It has been proposed that the mechanism changes fundamentally at this point and the oscillations are not compatible with consciousness. The second mechanism that will be addressed is a modification of the generally accepted mechanism for the spindle oscillations that occur during natural sleep (Steriade et al., 1993a,b). These two different mechanisms imply two different patterns for changes in the frequency of the thalamocortical oscillations during emergence. The first mechanism implies that the frequency of the oscillations should increase from the alpha range to the beta range during emergence. The “spindle” mechanism implies that the frequency of the oscillation would not increase much beyond the alpha range. Examples of EEG recordings during anesthesia and emergence from anesthesia were found which were consistent with either mechanism alone or both mechanisms at the same time. Neither theory was able to explain all examples. It is possible that both mechanisms can occur and that brainstem activity may influence the characteristics of emergence. The brainstem activity in question may be influenced by nociception and analgesic supplementation. It may be possible to control the path of emergence by controlling brainstem activity with opioids and other agents in order to allow the patient to awaken without going through an excitement phase or delirium at the transition to consciousness.

## Introduction

There are both practical and theoretical reasons to study emergence from anesthesia. Increasing the predictability of the time of emergence and reducing patient pain, delirium, or nausea after anesthesia are practical goals the professional anesthesia practitioner strives for their entire career without ever reaching perfection. The long elusive goal of understanding neurophysiologic mechanisms of anesthesia may be brought closer by tracking changes that occur during transitions into and out of anesthesia. Understanding the processes that occur during emergence may improve our ability to control it.

The hypothesis addressed here is that some elements of theories of mechanisms of anesthesia concerning oscillations in thalamic and cortical neuron systems can be evaluated with simple EEG analysis methods. For a theory of anesthesia mechanism based on thalamocortical oscillations to be valid, EEG data recorded in a *clinical setting* must be consistent with results from animal and computer model studies.

The approach that will be used herein to interpret the EEG derived data is fundamentally different from the approach that was used to develop currently available commercial EEG anesthesia monitors. Commercial EEG monitors produce a “depth of anesthesia” measure of general anesthesia which implies a model of brain function as a continuum from awake through anesthesia with differences that are largely quantitative. The assumption is that anesthetic agents impair brain function in a continuous graded manner. At some point a critical part of the unknown mechanism of consciousness fails. On commercially available EEG based anesthesia monitors responses to verbal stimuli stop, and presumably so does awareness, at approximately half way on the scale. Theoretically, the impairment of brain function continues until the EEG is isoelectric (Alkire, 1998; Rampil, 1998).

In contrast to the depth of anesthesia approach, the “thalamocortical oscillation” theory conceives that neuronal synchronization during consciousness is achieved through mechanisms that are fundamentally different than the synchronization of neurons during non-REM sleep and some forms of anesthesia. The oscillations during conscious states and the oscillations during unconscious states each occur in specific frequency ranges due to the kinetics of ion channels in the neurons that are involved. This abrupt change in the frequency and distribution of thalamocortical oscillations that occurs at the transition between states implies a different concept than progressive impairment continuing through loss of consciousness (Steriade et al., 1993a,b; Alkire et al., 2008; Mashour and Alkire, 2013; Purdon et al., 2015).

Using EEG data to evaluate theories of anesthesia mechanisms which are based on thalamocortical oscillations assumes that waves in the EEG and the peaks, troughs and other shape features of the EEG derived spectrum are determined by the kinetics of the ion channels that generate thalamocortical oscillations. Hence, they provide information concerning neurophysiology. The EEG analysis methods covered in this article are limited to methods that can be linked directly to the frequency and relative power of oscillations in EEG signals because it is hoped that these phenomena are linked to thalamocortical oscillations. This concept is at odds with the approach used by commercial EEG monitors. These monitors do not use shape features of the EEG spectrum directly to provide a measurement of the “depth of anesthesia”. The anesthesia indices produced by the algorithms employed by these monitors are not useful to evaluate theories of thalamocortical oscillations because many different EEG patterns will produce the same index value and there is no firm conceptual link between a series of e.g., state entropy (E-Entropy; GE Healthcare) or bispectral index (BIS™ Medtronic) values and changes in the frequency or power of any particular oscillation in neural systems.

EEG signals predominately reflect dendrite potentials on cortical pyramidal neurons which are the result of action potentials generated from cortical or thalamic neurons with axons that terminate on those dendrites. Brainstem activity is not directly detected in the EEG recorded at the scalp surface. However, thalamocortical oscillations are affected by brainstem activity due to changes in the membrane potential of thalamic and cortical neurons caused by neuromodulators projected from the brainstem. Changes in thalamocortical oscillations detected in the scalp surface EEG may sometimes be inferred to be caused by changes in brainstem activity.

### Theories Concerning Neural Systems and the EEG during Anesthesia

Two distinct theories of anesthesia mechanisms based on thalamocortical oscillations will be evaluated in this article. One theory is closely related to the mechanism for spindle waves during natural sleep. This mechanism has been extensively studied by neuroscientists but there have been few efforts to directly link this mechanism to clinical anesthesia. The other theory can be called the “traveling peak” theory which has been advanced by one group of researchers. It was developed specifically to explain EEG patterns produced during clinical anesthesia. Both mechanisms would produce similar oscillations in the alpha frequency range (8–12 Hz) within the EEG during surgical level anesthesia but those oscillations would move to different frequency ranges during emergence from anesthesia. Other theories concerning oscillations in EEG signals during anesthesia have been advanced but these two were chosen to illustrate an approach to evaluating such theories.

### “Traveling Peak” Mechanism

Theories of consciousness based on synchronization of 40 Hz oscillations have been proposed previously (Llinás and Ribary, 1993; Llinás et al., 1994). It is not unreasonable that 40 Hz oscillations are major components of the mechanism of consciousness but such theories remain incomplete. The “traveling peak” mechanism involves anesthetic agent effects on GABA receptors reducing the frequency of a 40 Hz oscillation that is generated in the cortex.

A study conducted with volunteers to whom propofol was administered in a stepwise manner and that tracked induction and emergence found that during sedation prior to loss of consciousness a peak in the EEG spectrum occurred in the beta frequency range (13–24 Hz). The frequency of this peak decreased as the level of propofol increased and entered the alpha frequency range (8–12 Hz) at loss of consciousness (Purdon et al., 2013). During emergence and the return of consciousness the frequency of the observed peak increased back into the beta range. The authors of this study named the peak the “traveling peak” because it changed frequency with propofol level. In this study, emergence was the reverse of induction in regard to the frequency shift in this “traveling peak”. Beta band activity with sedation and anesthesia induction has been reported before and has been established as a common phenomenon.

In an article published earlier by many of the same authors, a neurophysiologic mechanism for the “traveling peak” was proposed and tested with a computer model (Ching et al., 2010). The model and proposed mechanism described by Ching et al. (2010) involve a 40 Hz oscillation which occurs in cortical neurons prior to anesthesia. GABAergic inhibitory interneurons are critical to the cortical generation of the 40 Hz oscillation. As the level of propofol increases in the brain, GABA is potentiated at an increasing rate prolonging its inhibitory effects, which results in a decrease in the frequency of the oscillation. Ching et al. (2010) proposed that when the frequency decreases to the alpha range the cortical based oscillation entrains the thalamic neurons into the oscillation. The mechanism of consciousness continues to function but with continuously increasing impairment up until this point when the thalamus is entrained by the cortical rhythm. The entrainment of thalamic neurons includes the neurons of the reticular nucleus of the thalamus. Reticular thalamic neurons then synchronize with many thalamocortical neurons which in turn synchronize large areas of the cortex through the thalamocortical loop. The synchronization of large cortical areas is not compatible with consciousness. In theory, at that point the subject would not be able to process sensory input or stored memories at the level of the frontal cortex. This proposed mechanism will be referred to as the “traveling peak” mechanism.

In a later article (Vijayan et al., 2013) this proposed mechanism was expanded to include a second mechanism to explain the loss of occipital alpha rhythms. These rhythms are well known and occur only in the awake state with eyes closed and the subject relaxed. A third mechanism was proposed for the occurrence of frontal alpha rhythms which occur after the loss of consciousness with anesthesia as the occipital alpha oscillations end. The phenomenon of alpha rhythms stopping in the occipital cortex and beginning in the frontal cortex at loss of consciousness is also well known (John and Prichep, 2005). This observation has been termed “anteriorization of alpha”. Since occipital alpha rhythms end and frontal alpha rhythms begin approximate to the loss of consciousness, it may appear that the same mechanism causes both with just a shift in location. However, in reality they are two different mechanisms.

The second and third mechanisms (Vijayan et al., 2013) cited the effect of propofol on both GABA receptors and the voltage regulated ion channel, hyperpolarization-activated cation current “Ih” in thalamic neurons. The inhibition of Ih by propofol has been established (Cacheaux et al., 2005). The Ih channel is a nonspecific cation channel that predominantly increases sodium conductance. Hyperpolarization activates this channel which then depolarizes neurons shortening the duration of hyperpolarization induced by other ion channels. Anesthetic agents inhibit this channel causing thalamic neurons to remain longer at a hyperpolarized membrane potential. The model proposes that this action of anesthetic agents stops occipital cortex projecting thalamic neurons from producing action potentials in the alpha frequency range. Those neurons have ion channels which produce oscillations in the alpha range when depolarized and do not produce these oscillations when hyperpolarized. In contrast, frontal cortex projecting thalamic neurons have a different set of voltage dependant ion channels. Hyperpolarization caused by the inhibition of Ih will allow these thalamocortical and reticular thalamic neurons to activate each other in a “ping pong” manner. The frequency of this “ping pong” effect is in the alpha range because of the kinetics of the hyperpolarization activated voltage gated ion channels present in these neurons. The cortical “traveling peak” mechanism entrains thalamic neurons into their oscillation when the frequency decreases into the alpha range. Vijayan et al. (2013) adds to this concept of entrainment of a cortical based oscillation, an alpha oscillation intrinsic to the thalamus due to the “ping pong” effect between reticular thalamic and thalamocortical neurons.

The computer model of Vijayan et al. (2013) indicates that if the Ih channel is completely inhibited the alpha oscillations will not stop. The “ping pong” mechanism is similar to the mechanism of sleep spindles and may be considered a variation of the mechanisms proposed for sleep spindles. One major difference between the “ping pong” and the classic sleep spindle mechanism is that in the “ping pong” model the oscillation is created by thalamocortical and reticular thalamic neurons stimulating each other in a reciprocal fashion. The sleep spindle mechanism proposed by M. Steriade emphasized that the reticular thalamic neurons paced the oscillation because a surgically isolated reticular nucleus continued to produce the characteristic oscillation (Steriade et al., 1985, 1987). In subsequent publications by Steriade the mechanism allowed that there were significant effects on the oscillation by the interaction between cortical, thalamocortical and reticular thalamic neurons (Destexhe et al., 1998).

### Sleep Spindle Mechanism

It is well established that natural sleep produces spindle waves which are EEG oscillations that occur approximately within the alpha range (Loomis et al., 1935). Anesthesia with both propofol and inhaled halogenated agents also produces EEG oscillations in the alpha range. The relationship between anesthesia related frontal alpha oscillations and sleep related spindle oscillations has been reviewed (Sleigh et al., 2011). The experimental preparations that were used to develop the mechanism of sleep spindles most often involved some form of anesthesia. To date few detailed mechanisms that include specific types of neurons and ion channels have been advanced that would explain anesthesia induced alpha oscillations that do not either relate them to spindle oscillations produced during natural sleep or the mechanisms proposed in the studies previously mentioned in this article. This article will use EEG emergence data to evaluate the “traveling peak” and spindle mechanisms as they apply to anesthetized subjects. The sleep spindle mechanism applied to anesthesia will be referred to as the “spindle” mechanism. What follows is a summary of a widely accepted mechanism for sleep spindles (Steriade et al., 1993a,b; McCormick and Bal, 1997; Steriade, 2001; Traub et al., 2005; Crunelli et al., 2006).

Cortical pyramidal, thalamocortical and reticular thalamic neurons are three types of neurons that are required to produce spindle waves that are detectable in the EEG. Reticular neurons are GABAergic, mutually inhibit, and hence can synchronize each other. Since reticular neurons project to thalamocortical neurons, reticular neurons can synchronize thalamocortical neurons together with other reticular thalamic neurons. Thalamocortical neurons are excitatory and project to both pyramidal and reticular neurons. Some cortical pyramidal neurons project back to thalamocortical and reticular thalamic neurons exciting both and thus completing a loop. If the membrane potential of reticular and thalamocortical neurons are in a narrow range, hyperpolarized a few millivolts from their usual potential when awake, spindle waves may occur. In this membrane potential range, depolarizing input from corticothalamic or thalamocortical neurons will cause reticular thalamic neurons to depolarize and start cycles of depolarization and hyperpolarization at the frequency of spindle waves. In the depolarizing phase these neurons will fire bursts of action potentials. These action potentials will release GABA which will hyperpolarize thalamocortical neurons. The result is a rebound depolarization which may end with an action potential. This action potential produces dendrite potentials in pyramidal neurons at the frequency of spindle waves. The EEG primarily reflects dendrite potentials in pyramidal neurons since these neurons are oriented parallel to each other and perpendicular to the surface of the cortex. Reticular thalamic neurons determine the frequency of the spindle waves and can be loosely understood to be pacemakers for spindle waves.

The widely acknowledged concept that the membrane potential of both reticular thalamic and thalamocortical neurons must be in a narrow range of membrane potential for spindle oscillations to occur is related to a calcium channel (T channels) that both types of neurons possess. The T channel requires a period of hyperpolarization prior to depolarization in order for it to open. T channels can remain open and depolarize the neuron for a much longer period of time than depolarization produced as a result of a sodium channel based action potential. This prolonged depolarization can result in multiple action potentials instead of a single action potential. This is referred to as a “burst” and that the thalamocortical and reticular thalamic neurons have transitioned into “burst mode”.

The key points of this sleep spindle mechanism are that the frequency of the oscillation is determined largely by the reticular nucleus of the thalamus and does not produce oscillations substantially above or below the alpha range. All that is required to produce spindles is that thalamocortical and reticular thalamic neurons are in a particular range of membrane potential that is more negative than the membrane potential of these neurons when a subject is awake. This situation occurs during natural sleep when the brainstem projection of depolarizing neuromodulators such as acetylcholine, histamine and norepinephrine are reduced from levels that occur in subjects who are awake.

Anesthesia could change the membrane potential by any of several known effects. Possible mechanisms include potentiating GABA receptors, potentiating potassium ion channels, and/or inhibiting Ih ion channels in reticular thalamic and thalamocortical neurons (Banks and Pearce, 1999; Campagna et al., 2003; Grasshoff et al., 2006; Ying et al., 2006; Franks, 2008). The above mentioned mechanisms for change in membrane potential would be considered to be direct effects on thalamic neurons. Indirect mechanisms would include mimicking sleep by decreasing depolarizing projections from the brainstem to the thalamus.

There are differences between the characteristics of sleep spindles and anesthesia induced alpha oscillations. The major difference is that sleep spindles usually consist of sets of approximately a dozen oscillation cycles which are temporally separated from each other by gaps of several seconds. Alpha oscillations observed during anesthesia can have many more cycles in a set and the sets can occur less than 1 s apart. These differences may be explained by anesthetic agent inhibition of the Ih channel. It is thought that hyperpolarization during sleep spindles activates Ih which depolarizes thalamic neurons and terminates the spindle (Luthi and McCormick, 1998). During natural sleep this prevents another spindle for a few seconds. Models of sleep spindles and anesthesia induced alpha rhythms indicate that inhibition of Ih would result in continuous spindles (Vijayan et al., 2013).

### Anesthesia Transitioning to Natural Sleep during Emergence

It has been proposed that anesthesia could transition to natural sleep during the emergence process as an explanation for patient reports of dreaming during anesthesia (Leslie et al., 2009). Citing natural sleep as an explanation for dreaming prior to return of consciousness would mean a transition from anesthesia to non-REM and then REM sleep. This scenario would only be likely if there was a prolonged emergence without stimulation to arouse the subject. Dreaming occurs during REM sleep which begins after a period of non-REM sleep. Sleep spindles occur in non-REM, but not in REM sleep.

A study tracked the raw EEG of pediatric patients during emergence and monitored for delirium (Martin et al., 2014). Most subjects had EEG patterns during surgery that were dominated by “slow waves” in the alpha (8–12 Hz) and delta (1–4 Hz) ranges. The authors concluded that after the slow waves ended there often was a state between anesthesia and awareness. The authors called it the “indeterminate state”. If patients transitioned from the indeterminate state to EEG patterns similar to natural sleep they regained consciousness without delirium. Those who aroused in the “indeterminate state” were more likely to exhibit delirious behavior. Another study that included many of the same authors demonstrated emergence with arousal during alpha oscillations, or a transition to the indeterminate state prior to awareness (Hight et al., 2014). Some patients demonstrated an increase in the frequency of the alpha oscillations during emergence but some did not. One example increased the frequency of the alpha peak to 15 Hz and simultaneously developed a separate beta peak during emergence. Another example from the same study showed an increase in the frequency of the alpha peak to about 18 Hz and no other peak in the beta range A few patients never demonstrated slow wave anesthesia and possibly could be considered to be in the indeterminate state during surgery and emergence. Anesthetized patients that did not have alpha or delta EEG waves have been observed. This phenomenon has been termed “non slow wave anesthesia” (Chander et al., 2014) but no explanation is available.

### Brainstem Arousal System On-Off Switch

Natural sleep produces its characteristic EEG phenomena as a result of a reduction of brainstem induced depolarization of the thalamus and cortex (McCormick, 1989; McCormick et al., 1991; Fuller et al., 2006). There are multiple brainstem nuclei which project depolarizing neuromodulators to the thalamus and cerebral cortex. There is a nucleus in the brainstem that can affect the activity of all of these nuclei. This nucleus is the ventrolateral preoptic (VLPO) nucleus. When the VLPO is active (“on”) it projects GABA to multiple brainstem nuclei which reduce their projection of depolarizing neuromodulators to the thalamus and cortex. When the VLPO is less active (“off”) these brainstem nuclei are released from inhibition. These same nuclei also project back to the VLPO which acts to shut down the VLPO when the arousal nuclei are active. This relationship has been called a “flip flop switch” because there are sudden changes between on and off. There can be confusion since when the VLPO is “on” the brainstem arousal system is “off” due to active inhibition. If the VLPO is “off” the brainstem arousal system is released from active inhibition. To avoid confusion the phrase brainstem arousal system is “on” (or “off”) will be used instead of the VLPO is “off” or “on”.

The VLPO switch is thought to be important in the regulation of natural sleep (Saper et al., 2005). Studies have been conducted to determine whether the VLPO nucleus is involved in mechanisms of anesthesia (Lu et al., 2008; Li et al., 2009; Eikermann et al., 2011; Moore et al., 2012). In a study using rats as subjects, isoflurane increased activity in VLPO neurons but only one third as much as natural sleep (Lu et al., 2008). The membrane potential of cortical or thalamic neurons (i.e., level of hyperpolarization or depolarization) is the result of the sum of the directly acting hyperpolarizing effects of anesthetic agents and the depolarizing effects of the brainstem arousal system. If additional anesthetic agents such as opioids or dexmedetomidine could reduce the influence of the brainstem arousal system to the levels similar to that which occur in natural sleep, less anesthetic agent would be required to achieve the same degree of hyperpolarization of the thalamus and cortex.

If the “spindle” mechanism was in effect during anesthesia with very low activity of the brainstem arousal system, alpha oscillations would continue at low levels of anesthesia during emergence without much increase in frequency of the oscillation. If the brainstem arousal system was active during emergence the spindle mechanism would stop as the anesthetic agent level was reduced because the active brainstem arousal system would depolarize the thalamus and cortex. In that case the “traveling peak” mechanism could function and if it did the frequency of the alpha oscillation would increase well into the beta range unless the patient regained consciousness first.

The VLPO inhibits nuclei that project arousal neuromodulators. If the VLPO is “off” that only takes the “brakes” off which does not cause the arousal nuclei to become fully active (McCarren et al., 2014). Presumably, some stimulation must occur to cause the arousal system to become highly active. There is evidence that at reduced levels of anesthesia, activating the brainstem arousal system can result in emergence from anesthesia (McCarren et al., 2014) and inhibiting the arousal system delays emergence (McCarren et al., 2013).

If the brainstem arousal system activity was as low during anesthesia as it is during natural sleep and remained at that low level while anesthetic agents were eliminated, the thalamus and cortex would continue to be hyperpolarized despite anesthetic agent levels that were below the level where loss of response occurred during induction. At these low levels of anesthetic agent some effects from the agent would remain, but the effects related to low arousal system activity would become the dominant effect. This scenario would be required to occur for the subject to transition from anesthesia to a state indistinguishable from non-REM natural sleep.

### Differences between Proposed Mechanisms for Anesthesia and Emergence

There are some subtle but important differences between the previously mentioned “traveling peak” and “ping pong” mechanisms and mechanisms proposed by neuroscientists to explain the alpha band spindle rhythms of natural sleep. Analysis of EEG data from induction, maintenance and emergence may be able to help determine which mechanism is more consistent with the data. The “traveling peak” mechanism implies that the frequency of the alpha oscillation should change with changes in the effective amount of GABA potentiation over a wide range of anesthetic concentration. It also implies that alpha oscillations (8–12 Hz) should increase in frequency and thus become beta oscillations (13–24 Hz) during emergence. The “spindle” mechanism implies that the frequency of the alpha oscillation is determined primarily by the frequency of the oscillations in the reticular nucleus of the thalamus. Any change in frequency of the alpha oscillation is most likely primarily due to the result of effects at the reticular nucleus of the thalamus. The reticular nucleus of the thalamus has been studied extensively and there are no reports of it generating oscillations that would pace a spindle type effect in any frequency higher than the very low end of the beta range. Beta oscillations above baseline activity do not occur during natural sleep. Both the beta oscillations of the “traveling peak” and “spindle” oscillations could occur simultaneously and produce both beta and alpha oscillations in the EEG during anesthesia.

## Methods

In order to use the EEG signal to evaluate theories concerning oscillations of neural systems during emergence from anesthesia, it is necessary to process the signal in a manner so as to determine the amount of activity at different frequencies. The strength of oscillatory components in the signal can be presented using the power spectrum, i.e., as a power vs. frequency representation. This requires a transformation of the signal to the frequency domain. Or the EEG could be presented in the time domain where the single oscillatory components are extracted by adequate band pass filtering. The processed data can be displayed for visual inspection or analyzed by computer programs to report parameters. The fundamentals of EEG analysis for anesthesia have been reviewed (Rampil, 1998).

There are multiple ways to obtain the raw EEG signal from anesthetized patients. One method is to utilize the data port from a commercial device such as a BIS™ monitor. The routines used for signal processing in this manuscript were programmed in LabView or Matlab by the individuals listed in the acknowledgments. The data for this study was obtained almost 20 years ago from an Aspect A-1000 as part of an IRB approved clinical study. The electrodes where placed as recommended by the manufacturer at At1 and At2 with the reference at Fpz. After applying a notch filter at 60 Hz the signal was further band pass filtered to 0.25–47 Hz. The signal from the data port was in digital format with sampling at 128 Hz. This particular montage produces a limited view of EEG activity. It was adequate to detect sleep spindles in a subject who was sleeping in her usual fashion. The montage used to develop the “traveling peak” mechanism (Purdon et al., 2013) employed more electrodes, used a different reference electrode system, and covered a larger area of the scalp. How important those differences are for the purposes of evaluating theories of thalamocortical oscillations is not known.

### Time Domain Presentation of the EEG

**Figure 1:**
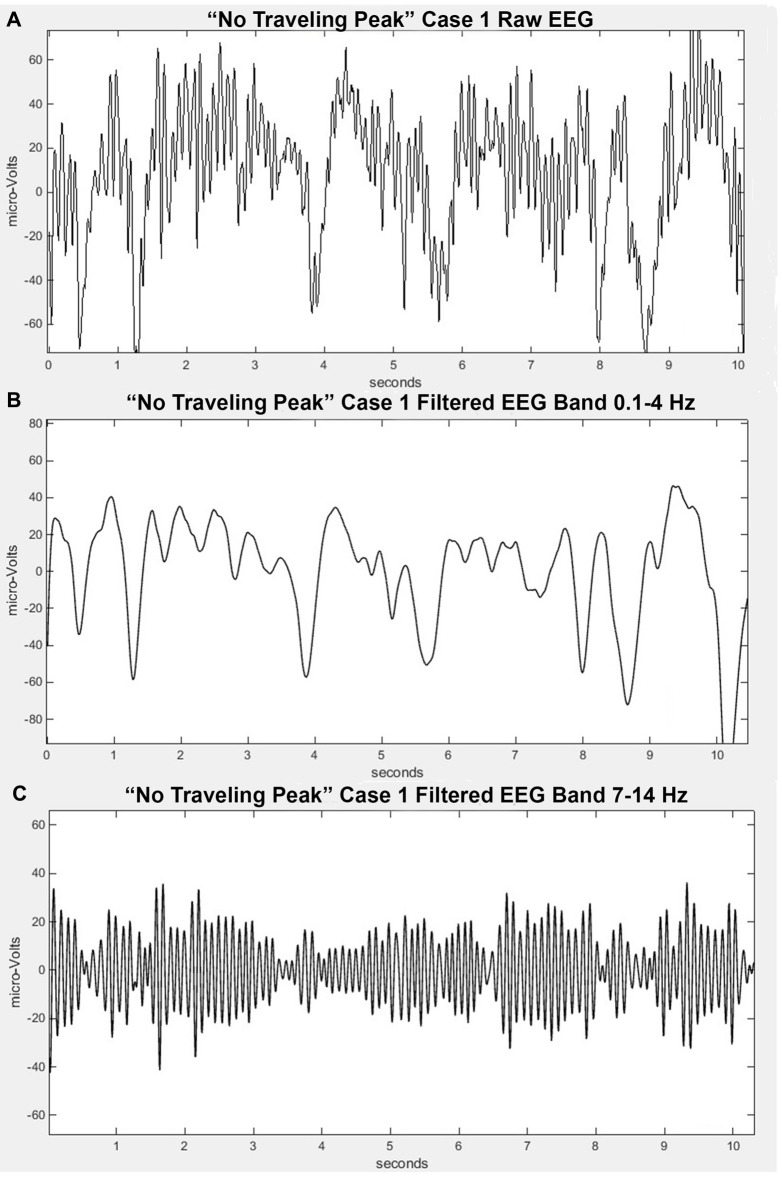
Filtered EEG signal from “No Traveling Peak” Case 1 at about the same time as in Figure 2. **(A)** is the “raw” signal. **(B)** is filtered to pass 0.1–4 Hz (delta). **(C)** is filtered to pass 7–14 Hz (alpha).

The EEG signal without any processing other than the filtering previously mentioned is commonly referred to by anesthesia clinicians as the “raw EEG”. An example appears in Figure 1A. This minimally processed EEG signal appears to be a mixture of oscillations at many different frequencies. If one inspects it carefully, one might notice that there are segments that might appear to be a series of waves at about 10 Hz and some large swings that could be in the range of 0.5–4 Hz. It has been suggested that anesthesia providers learn to interpret the “raw EEG” (Barnard et al., 2007; Bennett et al., 2009). In order to more clearly observe the high and low frequency signal dynamics, the EEG signals can be passed through a series of digital filters over frequency bands of interest and plotted on an appropriate scale. Figures 1B,C show examples of the EEG signal filtered from 7 Hz to 14 Hz and from 0.1 Hz to 4 Hz, respectively. The presentation of the time-domain waveform through a bank of band-pass filters has the advantage of visualizing details in the different bands that can potentially be missed when plotted on a large amplitude scale to accommodate the larger amplitude lower frequencies. This typically results in an improvement over attempting to decipher the level of a particular oscillation in the EEG signal.

### Frequency Domain Presentation of the EEG

**Figure 2:**
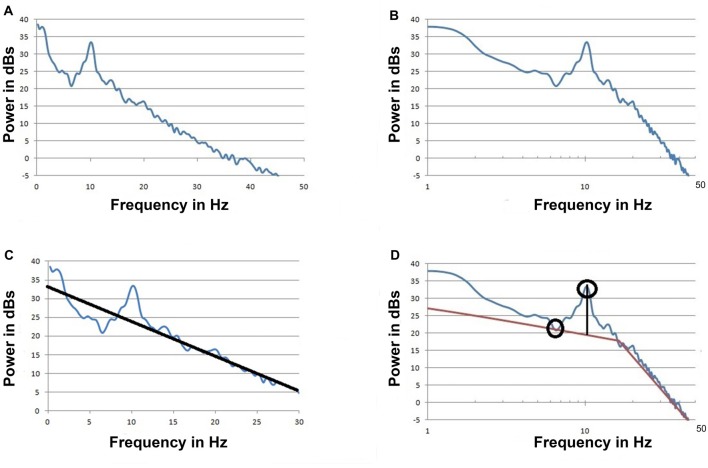
The spectrum of the signal from “No Traveling Peak” Case 1 at about the same time as the raw signal in Figure 1.** (A)** Power is on a log scale and frequency is on a linear scale (log-linear). **(B)** The same as **(A)** but with the frequency on a log scale (log-log) **(C)**. The same as **(A)** but with an approximation line to be used as a base line to measure the peak at 10 Hz. Data from 5 Hz to 7 Hz and 17 Hz to 35 Hz was used to draw the baseline as per Leslie et al. (2009). This graph displays the portion of the spectrum from 0 to 30 Hz only. **(D)** The same as** (B)** but with two approximation lines to be a base line for the peak. The maximum point of the peak and the minimum trough point are indicated by circles.

The signal can be (Fourier) transformed to frequency domain representation. The power spectrum that can be presented as a power vs. frequency graph is derived from the amplitude spectrum. Due to the large differences in power between the low frequency and high frequency ranges, the power is usually presented on a log scale. Typically the frequency is displayed on a linear scale (Figure 2A) but it can also be displayed on a log scale (Figure 2B). In this case it would be referred to as a log-log graph as opposed to the more common log-linear graph.

The power vs. frequency graph can display features such as peaks and troughs. A peak indicates more activity at that frequency while a trough indicates less. One approach to using the EEG to evaluate theories concerning the neurophysiology of anesthesia and emergence involves correlating oscillations at specific peaks in EEG spectra to the processes that are the basis of the theories. As the frequency and amplitude of the measured EEG oscillations change with anesthetic conditions, theories concerning neurophysiologic mechanisms are found to be either consistent with the data or not consistent. Tracking changes in the EEG over time can be accomplished with several techniques. This tracking can be done visually by combining multiple spectra. It also can be done by using a computer program to measure the features of the individual spectra and reporting a series of parameters.

There are well established methods for producing EEG based parameters such as the spectral edge frequency, spectral entropy, BIS and many others. None of these analytic methods would be useful to evaluate theories based on specific oscillations because they evaluate a wide frequency range. Power in specific bands such as alpha and beta may correlate with changes in oscillations in those bands but are not as useful for evidence of mechanisms as tracking peaks in spectra. If the peak shifted frequency within a band this may result in little or no change in the power within that band yet a change in frequency of the oscillation has occurred. Another problem with using the traditional EEG frequency bands is that the span of the alpha peak may not be entirely within the alpha band frequency range.

A method to measure the alpha peak above a baseline has been described (Leslie et al., 2009; Hight et al., 2014). Figure 2C illustrates this method. The height of peak above this baseline is thought to be due to oscillations that exceed background noise. Hight et al. (2014) used a log-linear spectrum with an upper limit of 30 Hz and a single approximation line to provide the baseline. When a log-log spectrum is used, two approximation lines are required as illustrated in Figure 2D. With either presentation a least squares approximation method is often used and eliminating the effects of peaks and artifacts is necessary. There is no perfect or standardized way to create the approximation lines.

Any method involving a baseline drawn by a computer program has potential for error related to changes in the shape of the spectra, shifting the baseline. This methodology could erroneously report a change in peak height above baseline when it was the computer rendering of the baseline that changed, not the activity that the peak represents. Another method involves using a computer program to detect a highest power peak point and compare that to a lowest power trough point (at a lower frequency). This method is illustrated in Figure 2D. With this method errors may occur if the program detects a “local minima” rather than a true trough point. Because of the possibility of such errors occurring, it is wise to check the underlying data with a visual method rather than to assume that all parameters reported by a computer program are accurate.

Another source for error or controversy in using a peak above baseline method is that as the process of emergence occurs the baseline may decrease as much as the maximum of the peak decreases. The “peak height” parameter would remain unchanged while the absolute amount of activity at that frequency declined. Since power is on a log scale what may appear to be a small change may in fact be a very large change on a linear basis. Because of these potential issues a visual method could be included to check the analysis.

## Results

A set of EEG recordings from an earlier IRB approved study was evaluated by visually examining the power spectra plots of those recordings. Power spectra with a peak in the alpha or beta range were selected for further evaluation with regard to changes in the frequency and power of alpha or beta peaks during emergence from anesthesia. Many of the recordings obtained during the study either did not have evidence for peaks or the recording was terminated soon after the completion of surgery and did not show EEG data in the emergence from anesthesia phase. Three patterns of peak behavior were observed in the recordings that did demonstrate peaks and examples of each are described below.

### Emergence without Change in Alpha Peak Frequency

**Figure 3:**
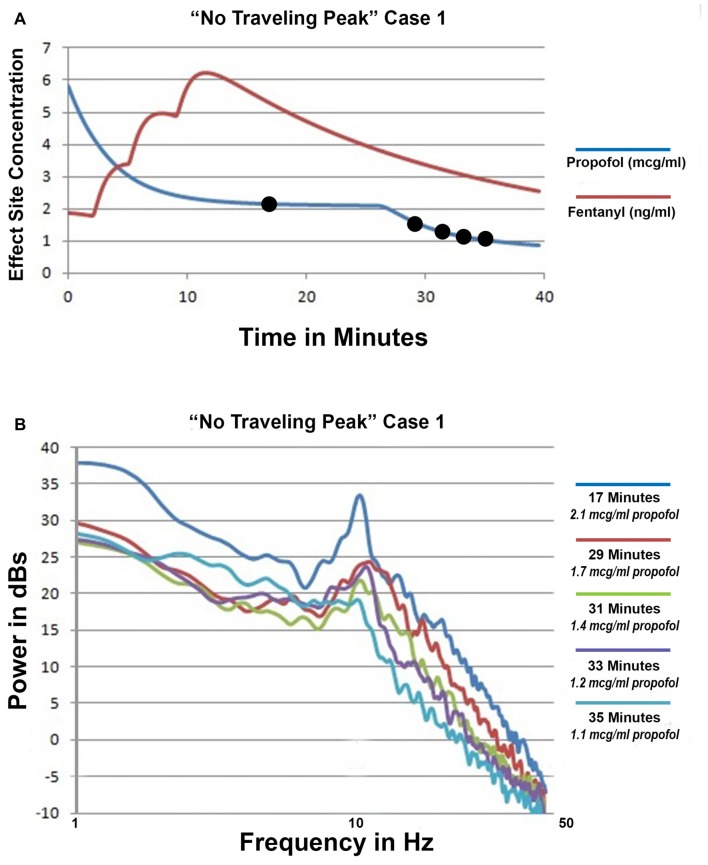
”No Traveling Peak” Case 1. **(A)** The calculated effect-site concentrations for propofol and fentanyl in mcg/ml and ng/ml, respectively. **(B)** Superimposed spectra from the times indicated by the legend.

**Figure 4:**
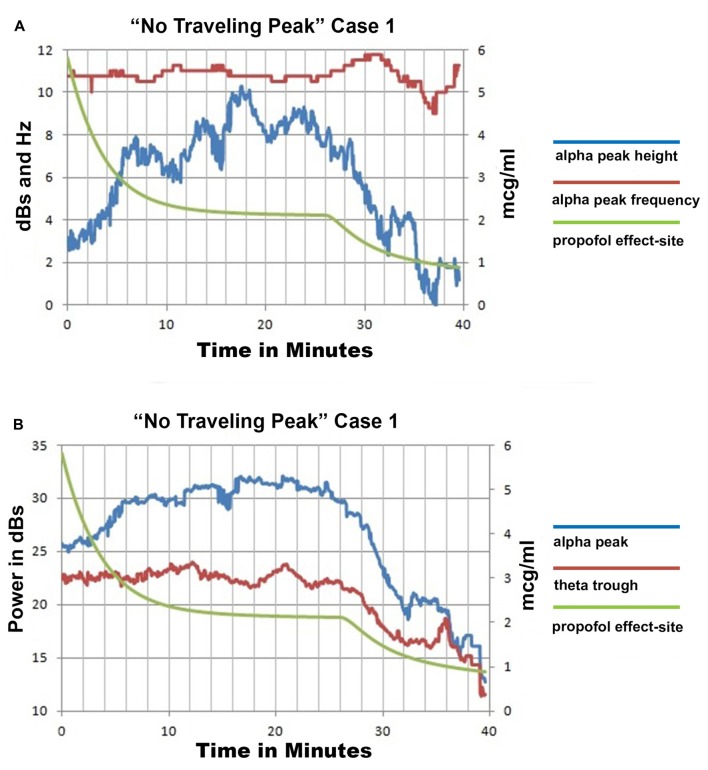
“No Traveling Peak” Case 1 EEG spectra based parameters and calculated effect site propofol concentration.** (A)** is the alpha peak center frequency and height above the trough point as illustrated in Figure 2D and the propofol effect-site concentration. **(B)** shows the power values for the peak and trough points as illustrated in Figure 2D and the propofol effect-site concentration.

**Figure 5:**
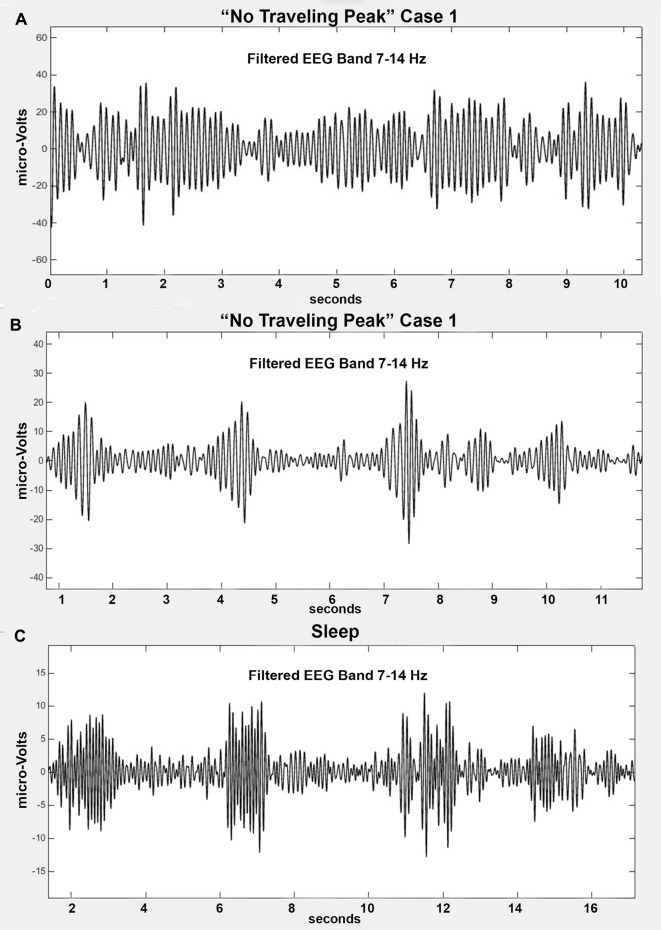
**(A)** Filtered alpha band from “No Traveling Peak” Case 1 at 17 min (2.1 mcg/ml effect-site propofol). This is the maximum amplitude of the alpha peak. **(B)** Filtered alpha band from “No Traveling Peak” Case 1 at 32 min which is 8 min after the propofol was discontinued (1.3 mcg/ml). **(C)** Filtered alpha band from an individual during non-REM sleep.

Only propofol and fentanyl anesthesia were used for “No Traveling Peak” Case 1. The surgical procedure was a diagnostic laparoscopy. The EEG episode in Figures 1A–C and the spectra in Figures 2A–D were generated in this case. Figure 3A shows the calculated effect-site concentrations for propofol and fentanyl and Figure 3B shows a series of spectra from “No Traveling Peak” Case 1. The spectrum (Figure 3B) at 17 min had the largest alpha peak during the procedure. The propofol infusion was discontinued at 26 min. The series of spectra indicate that there was a loss of power at all frequencies without a change in the frequency of the alpha peak during the process of emergence. The superimposed spectra in Figure 3B provide a visual presentation of the changes over time. A program was written in LabVIEW™ which tracks the frequency and amplitude of both the alpha peak maximum point and the theta trough minimum point. Those two points are indicated by circles in Figure 2D. Figure 4A shows that there are only small changes in the frequency of the alpha peak as the height of the peak above trough progresses to zero. Figure 4B indicates that in the 8 min after discontinuing the propofol infusion the alpha peak decreased by 10 decibels while the theta trough decreased by 5 decibels during the following 8 min. There was a decrease of power in all frequencies as the calculated propofol effect site concentration declined from 2.1 mcg/ml to 1.1 mcg/ml, but the alpha peak declined more and essentially disappeared. Figure 5A is the alpha filtered band at 17 min. The alpha frequency band oscillations are almost continuous. Figure 5B was at 32 min and shows 2 s gaps similar to Figure 5C which was another subject recorded during natural sleep. Figure 5C used a slightly different time scale in order to show an adequate number spindles. These results may indicate the possibility that the patient in “No Traveling Peak” Case 1 transitioned to a state comparable to non-REM natural sleep during emergence.

**Figure 6:**
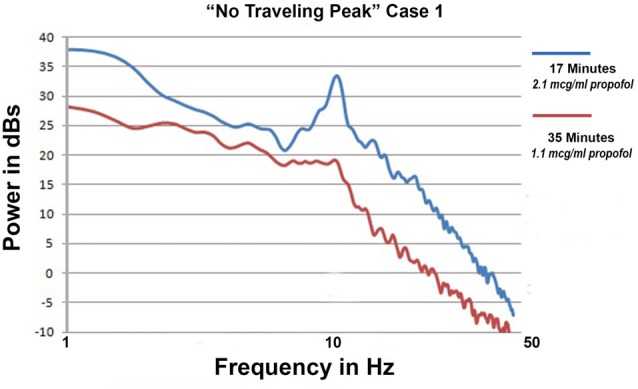
Two superimposed spectra from “No Traveling Peak” Case 1 at 17 and 35 min.

Figure 6 shows the spectrum at 35 min compared to 17 min. At 35 min there is not a significant peak anywhere. Inspection of the 7–14 Hz filtered EEG signal at 35 min did not find any oscillations that resembled sleep spindles. However, the shape of the spectra in a log-log presentation is similar to the shape of surgical anesthesia but at lower amplitude. The alpha peak is gone but the background activity is mostly unchanged. There appears to be a “spectral edge” at 10 Hz. At that frequency power diminished more rapidly with increasing frequency. These results raise the question as to whether there was a period of an “indeterminate state” or “non slow wave anesthesia” prior to the return of consciousness in this patient. The shape features of the log-log presentation may prove to be a useful tool to measure the “indeterminate state”.

At minute 35 the calculated effect site for propofol had declined to 1.1 mcg/ml while fentanyl was 3 ng/ml. Spontaneous respirations had not resumed at this point. Naloxone was administered IV and soon afterwards the patient was extubated and responding appropriately. Apparently the opioid was preventing the return of responsiveness even though the propofol level was low enough for appropriate responses to have occurred in the absence of the opioid.

### Alpha Peak Transition to Beta Peak during Emergence

**Figure 7:**
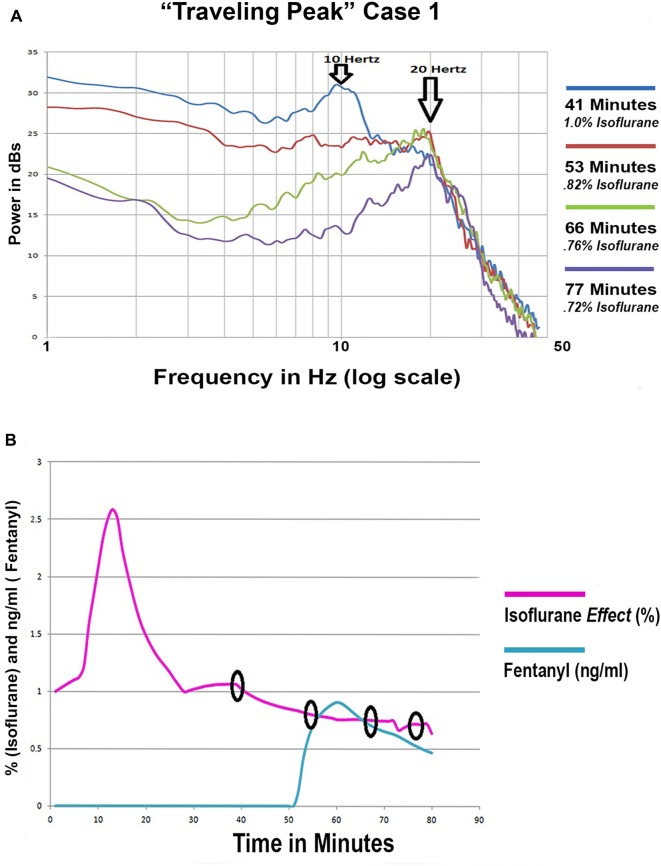
**(A)** Four superimposed spectra from “Traveling Peak” Case 1. **(B)** The calculated effect site concentration for fentanyl and isoflurane for “Traveling Peak” Case 1. A pharmacokinetic model was used for fentanyl. A transfer function adjustment of 20% per minute was used to convert measured end tidal isoflurane to effect site. The small black circles indicate the time when the spectra in 7 **(A)** were recorded.

“Traveling Peak” Case 1 involved a 36 year old female to whom isoflurane anesthesia was administered for a laparoscopic cholecystectomy. Superimposed spectra from “Traveling Peak” Case 1 are shown in Figure 7A. The calculated effect site concentration of both isoflurane and fentanyl are shown in Figure 7B. The times at which the 4 spectra were obtained are indicated by the circles in Figure 7B. This patient had a prominent alpha peak at 41 min. As the effect of the isoflurane was reduced (refer to Figure 7A) the center frequency of the peak increased to 19 Hz, well above the alpha range and clearly within the beta range as its amplitude decreased. Initially, a small “bump” appears in the beta range while the alpha peak is prominent. Later, as the alpha peak diminishes in amplitude the beta “bump” increases in amplitude and clearly becomes a peak. Careful inspection of the spectra reveals that perhaps the alpha peak did not morph into a beta peak but instead a separate and distinct beta peak formed as the alpha peak diminished in amplitude but is still present at the same alpha frequency. This observation may indicate that there are two different oscillations occurring with two different dose response relationships.

### Anesthesia with Both Alpha and Beta Peaks

**Figure 8:**
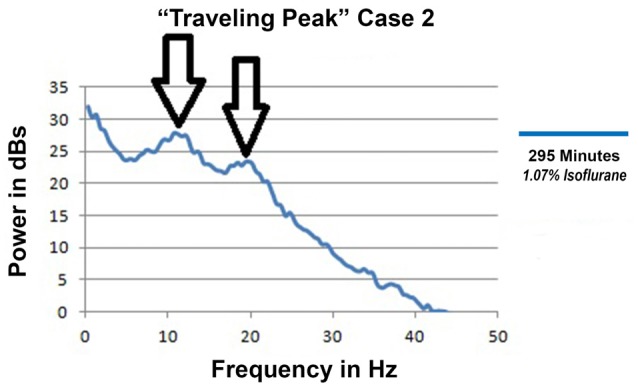
Spectra from “Traveling Peak” Case 2 at 295 min showing both an alpha and a beta peak.

“Traveling Peak” Case 2 was a 27 year old female to whom both isoflurane and epidural anesthesia were administered for an open reduction and internal fixation of a tibial fracture. Intravenous opioid was not used in this case. The EEG had distinct alpha and beta peaks which occurred simultaneously as illustrated in Figure 8. The double peak phenomena occurred and persisted during surgical anesthesia for more than 2 h with calculated effect site isoflurane concentrations between 1.5% and 1.03%.

## Discussion

The above noted anesthetic case examples were selected because they clearly showed three phenomena that are relevant to mechanisms of thalamocortical oscillations during anesthesia emergence. They are the loss of the alpha peak without much change in frequency, increasing frequency of the alpha peak, and both alpha and beta peaks appearing simultaneously. The anesthesia protocol was not designed to determine which factors, if any, may influence which of the three results will occur. There does appear to be a pattern as to the anesthesia factors and the EEG changes that were observed during emergence, but a firm correlation is not being claimed.

### Thalamocortical Oscillations during Anesthesia Emergence

The phenomenon of the alpha peak decreasing in amplitude without increasing in frequency seemed to occur most often when intravenous opioid was administered at levels high enough to result in very low respiratory rates or the absence of spontaneous respiration. These examples would be consistent with the “spindle” mechanism and the possibility of the brainstem arousal system being suppressed by opioid effects during emergence. If this phenomenon was due only to blocking of residual nociception from surgery, epidural anesthesia would produce the same effect. However, the cases with supplemental epidural anesthesia frequently behaved differently.

Whether or not this scenario can transition to a state closely resembling non-REM natural sleep remains possible but is not clearly proven by any of the cases reviewed.

The phenomenon of the alpha peak increasing in frequency during emergence appeared to occur most often with either epidural or lower doses of intravenous opioid. In many of those cases the alpha peak frequency never increased to above 15 or 16 Hz. It is possible that observation was achieved with a variation of the spindle mechanism and did not become the beta oscillation that is part of the “traveling peak” mechanism.

In other examples which demonstrated a clear beta peak during emergence, it appeared possible that a beta peak formed separately from the alpha peak. There were also examples with alpha and beta peaks clearly occurring at the same time and behaving independently. If the beta peak occurred simultaneously with the alpha peak and ended when the alpha peak ended it may be that some form of “harmonic” mechanism occurred which may be mostly a single mechanism. Perhaps there are two separate mechanisms but they become synchronized at harmonic multiples. If the beta peak continued after the alpha peak ended then they may have been caused by separate mechanisms.

The appearance of a beta peak cannot be explained by the “spindle” mechanism. There have been many articles published in the neuroscience literature regarding thalamocortical oscillations related to sleep spindles using a wide variety of experimental methods. There are no examples of sleep spindle related oscillations that can explain the “traveling peak” observation. A GABA effect on a cortical based 40 Hz rhythm which is the basis of the “traveling peak” mechanism is possible but is not the only possible explanation for a beta oscillation. There are 40 Hz oscillations generated in thalamic nuclei (Llinás and Ribary, 1993) and the same GABA effect could be at work there.

### Brainstem Activity and Thalamocortical Oscillations

Determining a level of opioid that would suppress the brainstem arousal system could be used to develop a strategy for the administration of anesthesia that would produce an emergence that would greatly reduce or possibly eliminate an excitement phase or delirium. An example of such a strategy might be as follows:

1. Anesthesia is induced in the usual fashion.
2. Intravenous opioid and the hypnotic agent (propofol or inhalation halogenated agent) levels are raised to a high enough level to likely put the brainstem in the mode of reduced projection of arousal neuromodulators (VLPO highly active).
3. Then the hypnotic agent is gradually reduced. Analgesia sufficient to prevent arousal is maintained and watched closely to prevent a decline below the critical level. Perhaps this might be achieved by a continuous infusion of remifentanyl.
4. Perhaps dexmedetomidine would be useful because of its unique effects on the brainstem arousal system.
5. Maintenance level of the hypnotic agent would be such that if the brainstem arousal system switched on the cortical and thalamic effects of the hypnotic agent would prevent awareness.
6. Monitoring the EEG spectra would likely be essential to verify that hypnotic and analgesic agent levels are appropriate. Near the end of the procedure the hypnotic agent level would be reduced further. After the last potentially painful element of the surgical procedure ends, the hypnotic agent should be discontinued.
7. No stimulation by the anesthesia provider should occur until the hypnotic agent’s cortical effect has diminished enough so that consciousness will return at brainstem arousal.

In summary, induction is designed to flip the brainstem arousal system to the “off” state. Then the analgesia level is closely followed to prevent flipping the brainstem arousal system to the “on” state until the end of the procedure. High levels of propofol or inhaled agent are not required to prevent consciousness once the brainstem switch is flipped to the “off” state. If patient responses to surgical stimulation result in movement, hypertension, or tachycardia and the EEG pattern does not show any change in the alpha band oscillations, these responses can be interpreted as spinal cord or autonomic nervous system reflexes and treated with agents specific to those reflexes rather than increasing the level of the inhaled anesthetic agent or propofol. If the thalamocortical oscillation theory is correct and brainstem arousal activity could be controlled during anesthesia maintenance and emergence, then employing this anesthesia strategy could in theory prevent intra-operative awareness, the excitement phase, and delirium during emergence.

The assertion that this strategy for the administration of an anesthetic could prevent intra-operative awareness, the excitement phase and delirium during emergence relies on a thalamocortical oscillation based theory of anesthesia and consciousness as opposed to the continuous incremental impairment theory of anesthesia as was mentioned in the introduction section. The thalamocortical oscillation based theory relies on synchronized oscillations linking widespread areas of the cortex. During consciousness this is accomplished by means of 40 Hz oscillations (Llinás and Ribary, 1993; Llinás et al., 1994). During non-REM sleep, spindle oscillations synchronize large cortical areas with 7–15 Hz oscillations in a manner that is not compatible with consciousness (Steriade et al., 1993a,b; Alkire et al., 2008; Mashour and Alkire, 2013; Purdon et al., 2015). The theory is that achieving and maintaining the anesthetized state involves a similar mechanism. The anesthetized state is not a lower “level of consciousness” but in contrast, no consciousness because the neurons are interacting in a manner that may be similar to how they interact during non-REM sleep and is considered to be incompatible with generating consciousness. The mechanism that produces switching between the two states is the change in membrane potential of thalamic neurons. Natural sleep and awareness switch back and forth due to brainstem activity changing the membrane potential of thalamic neurons. According to the theory, if an anesthetic regimen maintained the thalamus at a hyperpolarized membrane potential then intra-operative awareness would be prevented. If an anesthetic regimen could reduce brainstem arousal activity to a level similar to non-REM sleep and maintain that activity level while the inhaled agent or propofol levels were reduced to low enough levels, the subject would return to consciousness when brainstem activity increases to levels typical of consciousness. Excitement and delirium would occur only if the brainstem arousal system was active but unable to overcome the hyperpolarizing effects of residual anesthetic agents and reestablish consciousness.

## Conclusion

The loss of alpha peak amplitude without an increase in frequency cannot be explained by the “traveling peak” mechanism nor can beta peaks be explained by the “spindle” mechanism. Either mechanism could essentially be accurate for select individuals or when both peaks occur at the same time those individuals may have both mechanisms functioning simultaneously. The possibility of other mechanisms creating these EEG oscillation patterns has not been excluded.

The method of superimposing multiple spectra provided detailed information. The log-log presentation may prove superior to the log-linear presentation as it makes changes in the peaks and troughs easier to visualize. Comparing filtered bands was useful to verify peaks that appear in spectra. These are visual and hence subjective methods. Extracting measurements of peaks and troughs can provide objective evidence but should be checked to verify the validity of the extracted parameters.

The set of cases that provided the data examined in this article were not in themselves able to prove or disprove any neurophysiology based theory of anesthesia emergence. These observations were presented as an effort to demonstrate whether or not the phenomena postulated by the mechanisms that have been proposed can actually be observed during an anesthetic administered for a surgical procedure. The purpose of this hypothesis and theory article was to explain two mechanisms of thalamocortical oscillations that have been proposed as relevant to anesthesia and to illustrate a set of EEG analysis methods that if applied to a purposefully designed study might be able to provide evidence for or against such mechanisms. Such a study would likely show that either of the mechanisms may describe what may be observed in any given case. Detailing what factors determine which pattern occurs may be useful for clinicians to develop a strategy for anesthesia maintenance and emergence.

## Author Contributions

The research described herein is the sole work of CMS.

## Conflict of Interest Statement

The author declares that the research was conducted in the absence of any external funding. The author holds U.S. patent numbers 7,720,531, 8,352,021, and 8,401,631 which are related to processing and utilizing the EEG multiple approximation lines on a log-log spectrum (Figure 2D). Additional patents are pending.
